# Internet-Based Access to Sexual and Reproductive Health Services Among Colombian Youth: A Cross-Sectional Study

**DOI:** 10.1177/15404153241246102

**Published:** 2024-04-13

**Authors:** Julien Brisson, Karena D Volesky-Avellaneda

**Affiliations:** 1 Social and Behavioural Health Sciences Division, Dalla Lana School of Public Health, University of Toronto, Toronto, Ontario, Canada; 2 Department of Social and Preventive Medicine, School of Public Health, University of Montreal, Montreal, Quebec, Canada; 3 Department of Epidemiology, Biostatistics and Occupational Health, 5620 McGill University, Montreal, Quebec, Canada

**Keywords:** access, adolescent, Colombia, internet, sexual health, young people

## Abstract

**Introduction:** Sexual and reproductive health (SRH) is a sensitive subject, and young people may be unfamiliar with how to access SRH services. In this cross-sectional study, we examined young people's internet use to understand how they accessed SRH clinics in Colombia. This study also explored Colombian youth's interest in online material teaching how to access SRH services. **Methods:** During August 2019–February 2020, patients aged 10–24 years old presenting at two SRH clinics in Colombia were invited to answer a survey. Chi-square tests were performed to assess possible differences in how participants inquired how to access the clinic according to sociodemographic characteristics. **Results:** Among the 812 patients who participated, 91.4% were female and the median age was 19 years. To inquire how to access the SRH clinic, 30.7% of participants asked their parent(s) and 24.0% used the internet. Participants aged 20–24 years old were more likely to use the internet compared to younger participants (*p *< .001). Most respondents (81.5%) were interested in the availability of online material explaining how to access SRH services. **Conclusions:** While Colombian youth learned about how to access SRH clinics from several different sources, the vast majority indicated their interest in having access to online materials explaining how to access SRH services.

## Introduction

Globally, adolescents and young adults face numerous challenges when it comes to accessing sexual and reproductive health (SRH) services. These services encompass contraception, abortion, sexually transmitted infection (STI) testing and treatment, and related counseling (Decker et al., 2021; Morris & Rushwan, 2015; Pandey et al., 2019). Young people's limited awareness of available services and the requirements for utilization, such as costs or parental consent, exacerbate these obstacles (Newton-Levinson et al., 2016). The sensitive nature of SRH matters may lead young individuals to hesitate in seeking guidance from their parents or other family members. Existing literature highlights a strong preference for confidentiality and privacy when it comes to young people seeking SRH services (Brittain et al., 2015; Pampati et al., 2019; Schapiro & Mihaly, 2021). Moreover, even family members may lack awareness of the availability of such services and the pathways for young people to access them.

Recognizing the low utilization of health services among young people, the World Health Organization has advocated for public health initiatives that enhance adolescents’ access to healthcare services, including SRH services (WHO, 2017). This global health objective aligns with the United Nations’ *Sustainable Development Goals* concerning the promotion of adolescent health (UN, 2015). However, the available data on effective strategies to achieve this global health goal are limited (Brisson et al., 2021, 2024). Given young individuals’ widespread use of the internet for seeking health-related information, they may also use online sources to gather information on accessing local SRH services (Beck et al., 2014; Freeman et al., 2018, 2020; Magee et al., 2012). Yet, the literature provides scant information on this subject, especially regarding young people in developing countries (Robards et al., 2018).

The concept of young people's agency in navigating healthcare systems is underexplored, particularly among those residing in developing countries like Colombia (Bundy et al., 2018). In Colombia, where comprehensive sex education is not equally accessible in school settings (Albornoz-Arias et al., 2019; Brisson et al., 2023), understanding how young Colombians seek information on accessing SRH services is important. Furthermore, this topic is timely because the Colombian Health Ministry recently implemented initiatives to enhance young people's access to SRH services. These initiatives involve promoting youth-friendly health services that educate and empower young individuals to utilize SRH services, including contraceptives, STI testing, and HPV vaccination (Colombian Health Ministry, 2021).

The Colombian healthcare system is a complex mix of public and private health insurance. Young people can access certain health services (e.g., abortion) free of charge through their parents’ health insurance plans without their parents being notified (Brisson et al., 2021, 2024; Romero & Reingold, 2013). In other instances, there are publicly funded services that offer free services to young people, such as STI and HIV testing. Yet, young people in Colombia may underutilize SRH services, as observable with high rates of adolescent pregnancy and STI transmission (Encuesta Nacional de Demografía y Salud, 2015; Mercado & Sandoval, 2017; Murad-Rivera et al., 2018). For example, the proportion of pregnancies among adolescents aged 15–19 years old in Colombia increased from 13.0% in 1990 to 17.4% in 2015 (Profamilia, 2010, 2018; Uribe et al., 2010). During 2015–2021, Colombia saw a 21.5% reduction in births among adolescents aged 15–19 years old; however, in 2021, there were still 106,695 adolescents aged 15–19 years who gave birth (Departamento administrative nacional de estadistica – DANE, 2023). Hence, it is important to understand how young Colombian people access SRH services. Specifically, the factors that enable access, such as searching online. Initiatives focused on providing comprehensive youth-friendly online material explaining how to access such services may help improve SRH outcomes. Such material could include clear descriptions of the steps needed to obtain contraceptives and STI testing, clarify if there are fees for services, and confirm if confidentiality is respected.

The objective of this cross-sectional study was to reveal, among young Colombian people visiting a clinic specialized in SRH services, how they inquired to access the clinic, and to explore their interest in the availability of online material (e.g., videos) explaining how to access SRH services. The findings were compared across sexes, age groups, socioeconomic groups, and types of services sought at the clinic. In 2016, Colombia was one of two countries in Latin America, following Venezuela, where the majority of internet users were people aged 15–24 years old (López Ponce & Arcila Calderón, 2016). In 2021, 84.1% of 12–24-year-olds in Colombia were internet users (Ministerio de Tecnologías de la Información y las Comunicaciones, 2022). Given the substantial internet usage among young Colombian people, and its confidential nature, it was hypothesized that they predominantly utilize online platforms to inquire about accessing SRH services (Martin et al., 2020; Mustanski et al., 2023; Park & Kwon, 2018).

## Methodology

### Design

The study took place in two Profamilia clinics in the cities of Medellin and Cali (Colombia's second and third most populous cities after Bogotá). Profamilia is a network of nonprofit clinics across Colombia specializing in SRH and a member of the International Planned Parenthood Federation. Clinics in large city centers have “Youth Centers” specializing in the provision of services for people under age 25 (e.g., psychological counseling and teaching about safe sex practices).

From August 21, 2019 to February 14, 2020, receptionists invited patients aged 10–24 years old to answer the survey while they waited for their appointment. This extended age range represents the new definition of adolescence in global health (Sawyer et al., 2018). At Profamilia, young people requesting STI/HIV testing must fill out paperwork and discuss testing with a nurse. Since this process is already time consuming, patients requesting STI/HIV testing were not invited to answer the survey. Instead, the survey was specifically targeted at young patients accessing all other health services (e.g., abortion, contraceptives, sterilization procedures, HPV vaccinations). To avoid disrupting the quality of services offered, receptionists invited patients to participate during less busy periods. The invitation detailed the study's purpose and clarified that participation was voluntary (i.e., no remuneration) and that the quality of clinic services they received would not be influenced by whether they participated or not. Receptionists documented the number and dates that invitations were made thereby allowing us to quantify the level of participation. Participants’ anonymity was preserved—participants inserted the completed survey into a locked box that only the first author had access to.

The survey (in Spanish) contained three parts. The first part contained 28 multiple-choice questions designed to gather information on the participants’ autonomy and opinions about their access to SRH services. This section asked about the methods participants used to gather information about the clinic's services, and whether they were visiting the clinic alone or if they brought a companion. Additionally, participants were asked: *¿Qué tan interesado estarías en tener acceso a videos de YouTube y/o contenido en línea explicando en detalle cómo utilizar los servicios de salud sexual y reproductiva en tu comunidad?* (“How interested are you in accessing YouTube videos and/or online content that provides detailed explanations on using SRH services available in your community?”).

The second part of the survey contained 19 sociodemographic questions, including sex, age, *estrato* (socioeconomic classes assigned by the Colombian government based on residency; one is the lowest and six the highest), education level, and relationship status. The third part contained one open-ended question accompanied by a space for participants to provide their written response: *¿Hay algo que quieres agregar sobre el tema de la autonomía de los adolescentes para acceder a los servicios de salud sexual y reproductiva?* (“Is there anything you would like to add on the theme of adolescent autonomy in accessing SRH services?”). Since only seven participants provided responses related to the internet, a qualitative analysis was not conducted. Instead, we present these seven responses (translated from Spanish) to highlight participants’ viewpoints and to complement the quantitative data collected.

### Data Analysis

Participants were asked to select the ways they learned to access the clinic that day. It was possible to select more than one option. For instance, it was possible for a participant to have asked their friend and searched online. As such, data had to be recoded to help with data analysis and the responses were categorized based on the following priority order: (1) internet, (2) parents, (3) family members other than parents, and (4) friends or partners. For example, if the participant asked their father and their cousin, the answer was categorized once under the “parent” category and not in the “cousin” category. Given the different level of participation by age groups, the variable age was categorized as “younger adolescents” (11–16 years old), “older adolescents” (17–19 years old), and “young adults” (20–24 years old). Due to smaller counts, the upper three *estratos* were combined for analysis.

The four independent variables were sex, age group, *estrato*, and type of service sought at the clinic that day*.* We assessed whether there were statistically significant differences between the levels in each of the four independent categorical variables and how participants learned how to access the clinic (dependent variable coded as binary; e.g., internet vs all other sources) and second on their level of interest (very interested, interested, and not interest) in having online content explaining how to access SRH services. Statistical significance was assessed by performing Pearson chi-square tests of independence or Fisher's exact tests, as appropriate. *P*-values of <.05 were considered statistically significant, and 95% confidence intervals for proportions were calculated. Data analysis was performed in SPSS version 29 (IBM Corp, 2023).

### Ethics

The University of Montreal's Research Ethics Board (REB) first evaluated and approved the research project. Then, Profamilia's REB (which included a lawyer) evaluated and approved the research project. The REBs determined that the level of risk for participating was low. Parental consent was not required; it would have been challenging for patients who came alone to the clinic to obtain parental consent. Guideline 17 (Research Involving Children and Adolescents) from the *International Ethical Guidelines for Biomedical Research Involving Human Subjects* (Council for International Organizations of Medical Sciences, 2017) informed the decision to forego requesting parental consent. This guideline asserts that under specific conditions, the waiver of parental permission is permissible when obtaining it is neither feasible nor desirable, and when the research poses minimal risk to participants.

## Results

### Characteristics of Participants

Among 1,272 individuals invited to participate, 812 (63.8%) completed the survey. Seventy-three percent of participants answered the survey in Medellin's clinic (63.4% participation rate), and 27.0% in Cali's clinic (65.0% participation rate). Although there were more than two sex/gender options, all but six participants selected male or female (Table 1). Most participants were female (91.4%), reflecting the sex distribution of Profamilia patients in Colombia (Profamilia, 2018). There was a lower proportion of younger participants, and half were aged 16–20 years old (50.3%). Most participants were from the lower three *estratos* (86.0%), which is reflective of the distribution of the population in *estratos* across Colombia, where only a minority are in the upper three *estratos*. A tenth of participants were displaced because of violence, which is slightly less than the proportion of internally displaced people in Colombia (15.3% ∼7.7 out of 50.2 million people) (Reyes & Fattori, 2019). Most participants were Colombian (92.7%), a minority Venezuelan (1.7%), and the rest did not answer the question on nationality. The majority (75.9%) of participants aged 17 and younger reported being enrolled in school. Close to half (48.1%) of respondents aged 18 and older had attended postsecondary education.

**Table 1.** Characteristics of Survey Participants, *N* = 812.

Characteristic	Frequency	
	*n*	%
Sex		
Male	64	7.9
Female	742	91.4
Other	0	0.0
Missing	6	0.7
Age-group: years		
Mean (±SD)	18.6 (3.1)	
Median (IQR)	19 (16–21)	
Younger adolescents: 11–16	210	25.9
Older adolescents: 17–19	258	31.8
Young adults: 20–24	313	38.5
Missing	31	3.8
Socioeconomic class *(estrato)*^1^		
1 (lowest)	166	20.4
2	283	34.9
3	249	30.7
4	40	4.9
5	18	2.2
6 (highest)	3	0.4
Unknown	30	3.7
Missing	23	2.8
Internally displaced person^2^		
Yes	83	10.2
No	659	81.2
Missing	70	8.6
National status		
Colombian	753	92.7
Venezuelan	14	1.7
Missing	45	5.5
Relationship status		
Single	471	58.0
Serious relationship	251	30.9
Open union	31	3.8
Married	24	3.0
Missing	35	4.3
Enrolment in school (aged ≤17 years)^3^		
Enrolled	230	75.9
Not enrolled	66	21.8
Missing	7	2.3
Level of education (aged ≥18 years)^3^		
Postsecondary education	230	48.1
No postsecondary education	242	50.6
Missing	6	1.3

*Note:* SD = standard deviation; IQR = interquartile range.

1 *Estratos* are socioeconomic classes assigned by the Colombian government based upon one's residency.

2 Displaced people are individuals who had to relocate within a country because of violence.

3 Thirty-one participants did not provide their age, resulting in missing values for the education categories.

### Source of Information

There were 735 (90.5%) participants who provided one answer, 64 (7.9%) provided two answers and 13 (1.6%) provided three answers on the source of information they used to learn how to access the clinic (Table 2). Close to a quarter (24.0%; CI: 21.2%–27.0%) of participants used the internet to inquire about how to access the SRH clinic that day (Table 3), which was smaller than hypothesized. The most common source of information was “parent(s)” with 30.7% (CI: 27.6%–33.9%) having inquired to at least one parent on how to access Profamilia that day. Specifically, 27.4% responded “mother” and 2.3% “father” only (Table 2). Participants from the upper *estratos* were more likely to have used the internet to inquire about accessing Profamilia that day than those in lower *estratos* (*p *= .011). There were no significant differences between sexes in terms of sources, except that females were more likely to ask a family member (*p *= .010) or “other” sources (*p *= .027). Young adults (aged 20–24 years old) were more likely to use the internet to inquire on how to access the SRH clinic (*p *< .001).

**Table 2.** Sources Survey Participants Used to Learn How to Access Profamilia, Frequency, and Distribution.^1^

Source	Response	
	*n*	%
Mother only	205	22.7
Mother and other source(s)	42	4.7
Internet only	159	17.6
Internet and other source(s)	36	4.0
Referral^2^	119	13.2
Friend	110	12.2
Other family member	96	10.6
Learnt in school	54	6.0
Partner	22	2.4
Father only	21	2.3
Public advertisement	17	1.9
Missing	11	1.2
Other^3^	10	1.1
Total^4^	902	100.0

1 Participants were asked: “How did you know to come to Profamilia today to use the services? (More than one option possible)”

2 Referral from physician or from health insurance company.

3 Participants who selected “other” were asked to specify and provided the following answers: neighbor, teacher, social worker, prior experience with Profamilia.

4 Participant could choose more than one answer resulting in larger total number of answers compared to the number of participants.

**Table 3.** Sources Survey Participants Used to Learn How to Access Profamilia, by Sociodemographics and Type of Service Sought.

	Internet		Parent		Family member		Friend/partner		Referral		Other^6^		Missing		
Characteristic	*n*	%	*n*	%	*n*	%	*n*	%	*n*	%	*n*	%	*n*	%	Total^7^	
Sex															
Male	20	31.3	15	23.4	1	1.6	7	10.9	9	14.1	11	17.2	1	1.6	64
Female	171	23.0	233	31.4	86	11.6	79	10.6	98	13.2	65	8.8	10	1.3	742
*p* value	.139		.185		.010		.942		.847		.027		.600			
Age group^1^															
Younger adolescents	15	7.1	112	53.3	28	13.3	14	6.7	27	12.9	11	5.2	3	1.4	210
Older adolescents	76	29.5	77	29.8	31	12.0	25	9.7	27	10.5	20	7.8	2	0.8	258
Young adults	99	31.6	50	16.0	26	8.3	40	12.8	51	16.3	41	13.1	6	1.9	313
*p* value	<.001		<.001		.151		.073		.122		.006		.515			
Socioeconomic class *(estrato)*															
1 (lowest)	29	17.5	60	36.1	13	7.8	24	14.5	22	13.3	16	9.6	2	1.2	166
2	62	21.9	85	30.0	37	13.0	28	9.9	45	15.9	20	7.1	6	2.1	283
3	68	27.3	72	28.9	26	10.4	22	8.8	35	14.1	26	10.4	0	0.0	249
4–6 (highest)	24	39.3	18	29.5	5	8.2	3	4.9	3	4.9	8	13.1	0	0.0	61
*p* value	.003		.432		.318		.127		.162		.369		.093			
Type of service															
Abortion	11	35.5	4	12.9	4	12.9	7	22.6	1	3.2	3	9.7	1	3.2	31
Sterilization	18	34.0	11	20.8	3	5.7	5	9.4	5	9.4	10	18.9	1	1.9	53
Counseling/info	20	25.3	25	31.6	14	17.7	10	12.7	4	5.1	6	7.6	0	0.0	79
Contraceptives	131	21.9	201	33.6	62	10.4	56	9.3	94	15.7	49	8.2	6	1.0	599
Vaccination^2^	1	12.5	0	0.0	1	12.5	2	25.0	1	12.5	3	37.5	0	0.0	8
Pregnancy-related^3^	0	0.0	2	40.0	0	0.0	2	40.0	1	20.0	0	0.0	0	0.0	5
Other^4^	12	48.0	2	8.0	3	12.0	2	8.0	1	4.0	4	16.0	1	4.0	25
Missing	2	16.7	4	33.3	0	0.0	2	16.7	1	8.3	1	8.3	2	16.7	12
*p* value^5^	.012		.025		.282		.029		.014		.010		.730			
Total *n*	195		249		87		86		108		76		11			
% (95% CI)	24.0 (21.2–27.0)		30.7 (27.6–33.9)		10.7 (8.7–13.0)		10.6 (8.6–12.8)		13.3 (11.1–15.8)		9.4 (7.5–11.5)		1.4 (0.7–2.3)		812	

*Note:* CI = confidence interval.

1 Age was categorized as younger adolescents (11–16 years old), older adolescents (17–19 years old), and young adults (20–24 years old).

2 Included HPV and hepatitis B virus vaccination.

3 Pregnancy-related services included ultrasound, fertility problem, and assisted reproduction.

4 Other services included egg donation, polycystic ovaries, testicular pain, varicocele, pap smear, and menstrual pain.

5 Categories “other” and “missing” were excluded from the chi-square calculation.

6 “Other” responses included learned in school (*n* = 31), previous experience with Profamilia (*n* = 21), public advertisement (*n* = 14), and not specified (*n* = 10).

7 Missing responses to the sociodemographic questions (as reported in Table 1) were excluded from the row totals and tests of significance.

Table 3 also provides information on the type of service that the participant sought at Profamilia on the day they took the survey. The most frequently sought service was contraceptives (73.8%), followed by counseling and access to general SRH information (9.7%) and sterilization (6.5%). Note that sterilization can only be legally provided to individuals 18 years of age or older in Colombia. There were statistically significant differences for the categories: internet (*p *= .012), parent (*p *= .025), friend/partner (*p *= .029), referral (*p *= .014), and “other” (*p *= .010). 

### Interest in Online Content

Most participants indicated interest (81.5%; CI: 78.7%–84.1%) in having access to online material explaining how to access SRH services (Table 4). There were no statistically significant differences between sexes, *estratos* or type of service sought. Older participants were more likely to use the internet to inquire how to access Profamilia (Table 3), and they were more likely to be interested in having access to online material compared to younger participants.

**Table 4.** Participants’ Interest in Having Online Content Explaining How to Access SRH Services.^1^

	Very interested		Interested		Not interested		Missing			
Characteristic	*n*	%	*n*	%	*n*	%	*n*	%	*p* value^3^	Total^4^
Sex										
Male	16	25.0	38	59.4	10	15.6	0	0.0	.369	64
Female	233	31.4	370	49.9	133	17.9	6	0.8		742
Age group^2^										
Younger adolescent	51	24.3	110	52.4	46	21.9	3	1.4	.033	210
Older adolescent	74	28.7	134	51.9	47	18.2	3	1.2		258
Young adult	113	36.1	155	49.5	45	14.4	0	0.0		313
Socioeconomic class *(estrato)*		** **	** **	** **	** **	** **	** **	** **	** **	** **
1 (lowest)	54	32.5	77	46.4	35	21.1	0	0.0	.334	166
2	78	27.6	153	54.1	49	17.3	3	1.1		283
3	88	35.3	121	48.6	39	15.7	1	0.4		249
4–6 (highest)	16	26.2	35	57.4	10	16.4	0	0.0		61
Type of service										
Abortion	11	35.5	13	41.9	7	22.6	0	0.0	.756	31
Sterilization	20	37.7	22	41.5	11	20.8	0	0.0		53
Counseling/info	27	34.2	38	48.1	13	16.5	1	1.3		79
Contraceptives	177	29.5	314	52.4	105	17.5	3	0.5		599
Vaccination	2	25.0	4	50.0	1	12.5	1	12.5		8
Pregnancy-related	0	0.0	4	80.0	1	20.0	0	0.0		5
Other	12	48.0	9	36.0	3	12.0	1	4.0		25
Missing	2	16.7	7	58.3	3	25.0	0	0.0		12
Total, *n*	251		411		144		6			812
% (95% CI)	30.9 (27.8–34.2)		50.6 (47.2–54.0)		17.7 (15.2–20.5)		0.7 (0.27–1.6)			100.0

*Note:* CI - confidence interval.

1 Participants were asked: How interested are you in accessing YouTube videos and/or online content that provides detailed explanations on using sexual and reproductive health services available in your community?

2 Age was categorized as younger adolescents (11–16 years old), older adolescents (17–19 years old), and young adults (20–24 years old).

3 Categories “other” and “missing” were excluded from the chi-square calculation.

4 Missing responses to the socio-demographic questions (as reported in Table 1) were excluded from the row totals and tests of significance.

### Participants’ Comments on the Internet

In total, 113 (13.9%) participants provided feedback to the open-ended question. Participants’ comments touched on different themes, including parental consent, health fees, sex education in school, health professionals’ behavior, asking for free contraceptives, and stigma around sexuality. However, only seven (6.2%) of the 113 participants made comments related to the internet (Table 5). The comments suggest that accessing understandable information is a concern for some participants.

**Table 5.** Participants’ Responses Relating to the Internet for the Open-Ended Question.

Comments	Participant age and sex
**Importance to ask questions**	
*1. In my opinion, I would love that the information be made more accessible, for example, on good websites or blog where you can ask question about anything related to sex education. There are a few websites that exists, but they are very confusing.*	23-year-old female
**The need to access accurate information**	
*2. It would be very cool to receive information by email, see publicities online or social media. I think that way it would prevent us from being misinformed in many cases.*	24-year-old female
*3. I believe that it would be helpful to create a community (like webpages or YouTube videos, etc.) to make young people more interested [on the theme] and they can search a lot more information. Obviously, those pages should have a lot of true and accurate information on treatments like testing of HIV or sexual transmission (sic), confidentiality, etc.*	16-year-old female
**Interest in interacting with health professionals**	
*4. I would like that online, there’d be websites that share important information on sexual and reproductive health services. Also, it is important that in schools they teach us about places where we can access information about sex education. I would love that there’d be places to interact with professional people about the topic of sexual and reproductive health.*	18-year-old female
**The need for explanation**	
*5. I would like to access more easily a professional/expert on the theme to have a guide and help resolve the doubts that I have. Also, online material with images to better explain things.*	17-year-old female
*6. That there’d be different mediums of communications to give all the information to adolescents and adults*	23-year-old female
**Importance of discussing abortion**	
*7. I wish they could give discussions on abortion for adolescents, either online or at school.*	17-year-old female

## Discussion

The main source of information for participants to learn how to access SRH was their parents, predominantly mothers. This corresponds to findings in studies conducted in various regions, suggesting that adolescents tend to discuss sex-related matters more frequently with their mothers compared to their fathers (Scull et al., 2022; Usonwu et al., 2021; Wilson & Koo, 2010). Studies conducted in different cultural contexts have shown parents can be a main source to discuss general SRH with their adolescents (Jerman & Constantine, 2010; Evans et al., 2020; Rose et al., 2016). For example, Evans et al. (2020) reported that among 901 parents in the United States, 97% discussed topics about sexuality with their adolescent. This highlights the importance of ensuring parents possess adequate knowledge on informing their adolescents on *how* to access SRH services. While parents were the most commonly reported source, participants reported learning how to access SRH services from a diversity of sources, including friends, partners, other family members, and school. This diversity underscores the complexity of the networks through which youth navigate to obtain health-related information, highlighting the multifaceted nature of their efforts to access healthcare services.

Empirical literature reporting on young people's sources of information to inquire how to access health services is scarce (Amanu et al., 2023; Chekol et al., 2023). Most research has been conducted in high-income countries (Robards et al., 2018) and focused on young people's general health-seeking information behavior (Jia et al., 2022), as opposed to the source of information young people use to access health services.

The age span (10–24 years old) within our sample reflects the transition phase known as youth, where substantial developmental differences, particularly in independence, can be observed between individuals. Our results highlight substantial variations among these age groups, with older participants demonstrating a greater propensity to utilize the internet for inquiries about accessing the SRH services than their younger counterparts. Consequently, it would be beneficial for health education programs to tailor their approaches according to age.

A critical barrier young people face in utilizing SRH services is the lack of knowledge on how to access SRH services (Newton-Levinson et al., 2016). One measure to address the issue would be to include explanations to youth on how to access their local SRH services as part of comprehensive sex education programs in schools as highlighted in UNESCO's *International Technical Guidance on Sexuality Education* (UNICEF, 2018). However, in Colombia, young people do not have equal access to comprehensive sex education in schools (Mercado & Sandoval, 2017; Murad-Rivera et al., 2018), hence, the need for alternative measures to assist young people, such as through educational online material. Our research focused on Colombian youth attending SRH clinics, which means that these individuals received enough information to be able to access the clinic on the day they were surveyed. However, the broad spectrum of information sources utilized by them underscores the need for a nuanced understanding of information-seeking behaviors among Colombian youth. Future research should explicitly explore the various channels through which adolescents acquire information about accessing SRH services and validate its content. This measure is particularly important given the potential for youth in receiving inaccurate or insufficient information, which could subsequently hinder their ability to access these critical services.

Almost one-fifth of participants reported *no* interest in online content explaining how to access SRH services. Participants were not asked to explain the reasons behind their choice of answer; however, it is possible that they are not regular internet users or that they might prefer parents or other trusted individuals to explain how to access such services. Future research should investigate through which other means youth would like to be taught how to access SRH services.

Research in the United States has shown that young people can be distrustful of sexual health information found online and may prefer to obtain information in school or peers and parents (Cohn & Richters, 2013; Freeman et al., 2020; Jones & Biddlecom, 2011). Young people's use of the internet to search for health-related information represent different risks and opportunities (Comulada et al., 2021; Hollis et al., 2020; Jain & Bickham, 2014). The internet can be a useful source of information for young people, but misinformation is a risk (Madden et al., 2016). To help mitigate risks, public health initiates could explore integrating parents into online health initiatives for adolescents. With most participants (81.5%) showing interest in online resources for SRH service access, future public health efforts should aim to develop secure and reliable digital platforms that effectively engage youth in learning about SRH services, ensuring these platforms are designed to be informative and accessible to young people.

This study has multiple strengths. The sample size was large enough to provide meaningful insights into young people's experiences accessing SRH services in Colombia. Given the sensitivity of the subject matter, this survey achieved a respectable response rate of 63.8%. This may be attributed to the anonymity afforded to participants, which likely facilitated greater candor in responses. Furthermore, the study employed a range of response options to ensure participants could effectively communicate their experiences and perceptions, thereby enhancing the richness and depth of the data collected.

### Limitations

This study has limitations. First, the study sample was drawn from young people attending a SRH clinic for scheduled and walk-in appointments, which means they have already accessed SRH services and are as a result likely not representative of those who have not. For this reason, we caution readers in extrapolating the findings here to populations that have not accessed SRH services. Future research can explore the same topic with young people who have not accessed SRH services. Also, patients at Profamilia are not necessarily representative of patients who may access SRH services with their family physician or other clinics. While the survey garnered a respectable response rate, volunteer bias, where those who agree to participate and complete a survey differ from those who did not, may influence the results obtained. Originally, the recruitment approach was intended to be systematic, with receptionists inviting every patient between 10 and 24 years old to answer the survey. However, due to the workload of the receptionists, a convenience sampling approach was adopted, which may introduce selection bias. It is important to acknowledge that this change in sampling strategy could affect the representativeness of the sample. Furthermore, young patients present for HIV/STI testing were not invited to participate and they might have different answers on the subject. Finally, the survey did not undergo official piloting prior to dissemination.

## Conclusion

This study investigated how young individuals in Colombia access SRH services, focusing on their use of the internet. Findings showed that only 24.0% of participants utilized the internet for this purpose, while 30.7% relied on their parents as a primary source of information (mostly mothers), indicating the critical role of parents in helping young people to access to SRH services. However, 81.5% expressed interest in accessing online resources to learn about accessing SRH services. These findings highlight the importance of parents and the internet in improving the accessibility of SRH services for young people in Colombia and should be considered by healthcare professionals and policymakers.

## References

[bibr1-15404153241246102] Albornoz-Arias N. Mazuera-Arias R. Carreño-Paredes M. T. Vivas-García M. Cuberos M. A. Ramírez-Martínez C. Ortíz F. A. Bermúdez V. (2019). Influencia de la educación sexual en la maternidad adolescente en el Departamento Norte de Santander, Colombia. Archivos Venezolanos de Farmacología y Terapéutica, 38(1), 82–91.

[bibr2-15404153241246102] Amanu A. Birhanu Z. Godesso A. (2023). Sexual and reproductive health literacy among young people in sub-Saharan Africa: Evidence synthesis and implications. Global Health Action, 16(1), 2279841. 10.1080/16549716.2023.2279841 38010100 PMC10795590

[bibr3-15404153241246102] Beck F. Richard J. B. Nguyen-Thanh V. Montagni I. Parizot I. Renahy E. (2014). Use of the internet as a health information resource among French young adults: Results from a nationally representative survey. Journal of Medical Internet Research, 16(5), e128. 10.2196/jmir.2934 PMC4051740 24824164

[bibr4-15404153241246102] Brisson J. Ravitsky V. Williams-Jones B. (2021). “Fostering autonomy” for adolescents to access health services: A need for clarifications. Journal of Adolescent Health, 68(6), 1038–1039. 10.1016/j.jadohealth.2021.03.007 34023061

[bibr5-15404153241246102] Brisson J. Ravitsky V. Williams-Jones B. (2023). Colombian Youth express interest in receiving sex education from their parents. Sexuality & Culture, 27(1), 266–289. 10.1007/s12119-022-10012-8

[bibr6-15404153241246102] Brisson J. Ravitsky V. Williams-Jones B. (2024). Colombian Adolescents’ perceptions of autonomy and access to sexual and reproductive health care services: An ethical analysis. Journal of Adolescent Research, 39(2), 298–327. 10.1177/07435584211014862

[bibr7-15404153241246102] Brittain A. W. Williams J. R. Zapata L. B. Moskosky S. B. Weik T. S. (2015). Confidentiality in family planning services for young people: A systematic review. American Journal of Preventive Medicine, 49(2), S85–S92. 10.1016/j.amepre.2015.04.001 PMC4579525 26190851

[bibr8-15404153241246102] Bundy D. A. de Silva N. Horton S. Patton G. C. Schultz L. Jamison D. T. Abubakara A. Ahuja A. Alderman H. Allen N. Appleby L. Aurino E. Azzopard P. Baird S. Banham L. Behrman J. Benzian H. Bhalotra S. Bhutta Z. Black M. Bloem P. Bonell C. Bradley M. Brinkman S. Brooker S. Burbano C. Burnett N. Cernuschi T. Clarke S. Coffey C. Sawyer S. M. (2018). Investment in child and adolescent health and development: Key messages from disease control priorities. The Lancet, 391(10121), 687–699. 10.1016/S0140-6736(17)32417-0 29153316

[bibr9-15404153241246102] Chekol B. M. Sheehy G. Siraneh Y. (2023). Sexual and reproductive health experiences, access to services, and sources of information among university students in Ethiopia. Frontiers in Reproductive Health, 5, 1271685. 10.3389/frph.2023.1271685 38162013 PMC10755960

[bibr10-15404153241246102] Cohn A. Richters J. (2013). My vagina makes funny noises’: Analyzing online forums to assess the real sexual health concerns of young people. International Journal of Sexual Health, 25(2), 93–103. 10.1080/19317611.2012.719852

[bibr11-15404153241246102] Colombian Health Ministry. (2021). Derechos sexuales y reproductivos para adolescentes y Jovenes. Retrieved May 28, 2021, from https://www.minsalud.gov.co/salud/publica/ssr/Paginas/Derechos-sexuales-y-reproductivos-para-adolescentes-y-jovenes.aspx

[bibr12-15404153241246102] Comulada W. S. Goldbeck C. Almirol E. Gunn H. J. Ocasio M. A. Fernández M. I. Arnold E. M. Romero-Espinoza A. Urauchi S. Ramos W. Rotheram-Borus M. J. Klausner J. D. Swendeman D. , & Adolescent Medicine Trials Network (ATN) CARES Team. (2021). Using machine learning to predict young people’s internet health and social service information seeking. Prevention Science, 22(8), 1173–1184. 10.1007/s11121-021-01255-2 33974226 PMC8541921

[bibr13-15404153241246102] Council for International Organizations of Medical Sciences (2017). International Ethical Guidelines for Health-related Research Involving Humans (4th ed.). WHO Press. 40523065

[bibr14-15404153241246102] Decker M. J. Atyam T. V. Zárate C. G. Bayer A. M. Bautista C. Saphir M. (2021). Adolescents’ perceived barriers to accessing sexual and reproductive health services in California: A cross-sectional survey. BMC Health Services Research, 21, 1–12. 10.1186/s12913-021-07278-3 34809640 PMC8609799

[bibr15-15404153241246102] Departamento administrative nacional de estadistica— DANE . (2023). Nacimientos en niñas y adolescentes en Colombia (segunda edición). Retrieved February 21, 2023, from https://www.dane.gov.co/files/investigaciones/notas-estadisticas/ago_nota-estadistica-embarazo-020822_VF.pdf

[bibr16-15404153241246102] Encuesta Nacional de Demografía y Salud. (2015). Componente de Salud Sexual y Salud Reproductiva : Tomo II. *Ministerio de la salud de Colombia*. https://profamilia.org.co/wp-content/uploads/2019/05/ENDS-2015-TOMO-II.pdf

[bibr17-15404153241246102] Evans R. Widman L. Kamke K. Stewart J. L. (2020). Gender differences in parents’ communication with their adolescent children about sexual risk and sex-positive topics. The Journal of Sex Research, 57(2), 177–188. 10.1080/00224499.2019.1661345 31517541

[bibr18-15404153241246102] Freeman J. L. Caldwell P. H. Bennett P. A. Scott K. M. (2018). How adolescents search for and appraise online health information: A systematic review. The Journal of Pediatrics, 195, 244–255. 10.1016/j.jpeds.2017.11.031 29398062

[bibr19-15404153241246102] Freeman J. L. Caldwell P. H. Scott K. M. (2020). The role of trust when adolescents search for and appraise online health information. The Journal of Pediatrics, 221, 215–223. 10.1016/j.jpeds.2020.02.074 32446485

[bibr20-15404153241246102] Hollis C. Livingstone S. Sonuga-Barke E. (2020). The role of digital technology in children and young people's mental health–a triple-edged sword? Journal of Child Psychology and Psychiatry, 61(8), 837–841. 10.1111/jcpp.13302 32706126

[bibr21-15404153241246102] IBM Corp (2023). IBM SPSS Statistics for Macintosh, version 29.0.

[bibr22-15404153241246102] Jain A. V. Bickham D. (2014). Adolescent health literacy and the internet: Challenges and opportunities. Current Opinion in Pediatrics, 26(4), 435–439. 10.1097/MOP.0000000000000119 24886952

[bibr23-15404153241246102] Jerman P. Constantine N. A. (2010). Demographic and psychological predictors of parent–adolescent communication about sex: A representative statewide analysis. Journal of Youth and Adolescence, 39, 1164–1174. 10.1007/s10964-010-9546-1 20458614 PMC2917005

[bibr24-15404153241246102] Jia R. M. Du J. T. Zhao Y. C. (2022). Characteristics of the health information seeking behavior of LGBTQ+ individuals: A systematic review on information types, information sources and influencing factors. Journal of Documentation, 78(2), 361–388. 10.1108/JD-03-2021-0069

[bibr25-15404153241246102] Jones R. K. Biddlecom A. E. (2011). Is the internet filling the sexual health information gap for teens? An exploratory study. Journal of Health Communication, 16(2), 112–123. 10.1080/10810730.2010.535112 21207311

[bibr26-15404153241246102] López Ponce M. Arcila Calderón C. (2016). Adopción y uso de medios sociales por jóvenes de la costa caribe de Colombia. Investigación y Desarrollo, 24(2), 285–306. 10.14482/indes.24.2.8906

[bibr27-15404153241246102] Madden T. Cortez S. Kuzemchak M. Kaphingst K. A. Politi M. C. (2016). Accuracy of information about the intrauterine device on the internet. American Journal of Obstetrics and Gynecology, 214(4), 499-e1–499.e6. 10.1016/j.ajog.2015.10.928 PMC4808607 26546848

[bibr28-15404153241246102] Magee J. C. Bigelow L. DeHaan S. Mustanski B. S. (2012). Sexual health information seeking online: A mixed-methods study among lesbian, gay, bisexual, and transgender young people. Health Education & Behavior, 39(3), 276–289. 10.1177/1090198111401384 21490310

[bibr29-15404153241246102] Martin P. Cousin L. Gottot S. Bourmaud A. de La Rochebrochard E. Alberti C. (2020). Participatory interventions for sexual health promotion for adolescents and young adults on the internet: Systematic review. Journal of Medical Internet Research, 22(7), e15378. 10.2196/15378 PMC7428916 32735217

[bibr30-15404153241246102] Mercado C. A. G. Sandoval G. M. (2017). Prevalencia de embarazo y características demográficas, sociales, familiares, económicas de las adolescentes, carepa, Colombia. CES Salud Pública, 8(1), 25–33.

[bibr31-15404153241246102] Ministerio de Tecnologías de la Información y las Comunicaciones. (2023). Colombia avanza en su meta de estar conectada en un 70% en 2022: DANE. Retrieved January 11, 2023, from https://mintic.gov.co/portal/inicio/Sala-de-prensa/182108:Colombia-avanza-en-su-meta-de-estar-conectada-en-un-70-en-2022-DANE

[bibr32-15404153241246102] Morris J. L. Rushwan H. (2015). Adolescent sexual and reproductive health: The global challenges. International Journal of Gynecology & Obstetrics, 131(S1), S40–S42. 10.1016/j.ijgo.2015.02.006 26433504

[bibr33-15404153241246102] Murad-Rivera R. Rivillas-García J. C. Victoria V. Forero-Martínez L. J. (2018). Determinantes del embarazo en adolescentes en Colombia: Explicando las causas de las causas. Profamilia. 10.13140/RG.2.2.22412.95362

[bibr34-15404153241246102] Mustanski B. Saber R. Macapagal K. Matson M. Laber E. Rodrgiuez-Diaz C. Moran K. O. Carrion A. Moskowitz D. A. Newcomb M. E. (2023). Effectiveness of the SMART sex ed program among 13–18 year old English and Spanish speaking adolescent men who have sex with men. AIDS and Behavior, 27(2), 733–744. 10.1007/s10461-022-03806-2 35951143 PMC10249335

[bibr35-15404153241246102] Newton-Levinson A. Leichliter J. S. Chandra-Mouli V. (2016). Sexually transmitted infection services for adolescents and youth in low-and middle-income countries: Perceived and experienced barriers to accessing care. Journal of Adolescent Health, 59(1), 7–16. 10.1016/j.jadohealth.2016.03.014 PMC5289742 27338664

[bibr36-15404153241246102] Pampati S. Liddon N. Dittus P. J. Adkins S. H. Steiner R. J. (2019). Confidentiality matters but how do we improve implementation in adolescent sexual and reproductive health care? Journal of Adolescent Health, 65(3), 315–322. 10.1016/j.jadohealth.2019.03.021 PMC8130220 31227388

[bibr37-15404153241246102] Pandey P. L. Seale H. Razee H. (2019). Exploring the factors impacting on access and acceptance of sexual and reproductive health services provided by adolescent-friendly health services in Nepal. PLoS One, 14(8), e0220855. 10.1371/journal.pone.0220855 PMC6687105 31393927

[bibr38-15404153241246102] Park E. Kwon M. (2018). Health-related internet use by children and adolescents: Systematic review. Journal of Medical Internet Research, 20(4), e120. 10.2196/jmir.7731 PMC5904452 29615385

[bibr39-15404153241246102] Profamilia. (2010). Encuesta Nacional de Demografía y Salud: 2010. Bogotá. Retrieved March 8, 2023, from https://dhsprogram.com/pubs/pdf/fr246/fr246.pdf

[bibr40-15404153241246102] Profamilia. (2018). Informe de Gestion 2018. Retrieved February 21, 2023 from https://profamilia.org.co/wp-content/uploads/2019/06/Profamilia-informe-gestion-2018.pdf

[bibr41-15404153241246102] Reyes L. A. D. Fattori G. (2019). Microfinance as a means for women empowerment in the Colombian post conflict scenario: Transformational development or a tool for better managing poverty? Peace Human Rights Governance, 3(1), 127–161. 10.14658/pupj-phrg-2019-1-5

[bibr42-15404153241246102] Robards F. Kang M. Usherwood T. Sanci L. (2018). How marginalized young people access, engage with, and navigate health-care systems in the digital age: Systematic review. Journal of Adolescent Health, 62(4), 365–381. 10.1016/j.jadohealth.2017.10.018 29429819

[bibr43-15404153241246102] Romero K. Reingold R. (2013). Advancing adolescent capacity to consent to transgender-related health care in Colombia and the USA. Reproductive Health Matters, 21(41), 186–195. 10.1016/S0968-8080(13)41695-6 23684201

[bibr44-15404153241246102] Rose I. D. Friedman D. B. Spencer S. M. Annang L. Lindley L. L. (2016). Health information–seeking practices of African American young men who have sex with men: A qualitative study. Youth & Society, 48(3), 344–365. 10.1177/0044118X13491769

[bibr45-15404153241246102] SawyerS. M. AzzopardiP. S. WickremarathneD. PattonG. C. (2018). The age of adolescence. The Lancet Child & Adolescent Health, 2(3), 223–228. https://doi.org/S2352-4642(18)30022-130169257

[bibr46-15404153241246102] Schapiro N. A. Mihaly L. K. (2021). The 21st century cures act and challenges to adolescent confidentiality. Journal of Pediatric Health Care, 35(4), 439–442. 10.1016/j.pedhc.2021.03.005 33865680

[bibr47-15404153241246102] Scull T. M. Carl A. E. Keefe E. M. Malik C. V. (2022). Exploring parent-gender differences in parent and adolescent reports of the frequency, quality, and content of their sexual health communication. The Journal of Sex Research, 59(1), 122–134. 10.1080/00224499.2021.1936439 34114908 PMC9594984

[bibr48-15404153241246102] UNICEF (2018). International technical guidance on sexuality education: an evidence-informed approach. UNESCO Publishing.

[bibr49-15404153241246102] United Nations (2015). UN General Assembly Transforming our World: The 2030 Agenda for Sustainable Development. United Nations.

[bibr50-15404153241246102] Uribe A. F. Orcasita L. T. Vergara Vélez T. (2010). Factores de riesgo para la infección por VIH/SIDA en adolescentes y jóvenes colombianos. Acta Colombiana de Psicología, 13(1), 11–24. 10.14658/pupj-phrg-2019-1-5

[bibr51-15404153241246102] Usonwu I. Ahmad R. Curtis-Tyler K. (2021). Parent–adolescent communication on adolescent sexual and reproductive health in sub-saharan Africa: A qualitative review and thematic synthesis. Reproductive Health, 18(1), 1–15. 10.1186/s12978-021-01246-0 34629082 PMC8504018

[bibr52-15404153241246102] Wilson E. K. Koo H. P. (2010). Mothers, fathers, sons, and daughters: Gender differences in factors associated with parent-child communication about sexual topics. Reproductive Health, 7(1), 1–9. 10.1186/1742-4755-7-31 21156057 PMC3019147

[bibr53-15404153241246102] World Health Organization (2017). Global Accelerated Action for the Health of Adolescents (AA-HA!): Guidance to support country implementation. World Health Organization.

